# An ARFID case report combining family-based treatment with the unified protocol for Transdiagnostic treatment of emotional disorders in children

**DOI:** 10.1186/s40337-019-0267-x

**Published:** 2019-10-24

**Authors:** Sarah Eckhardt, Carolyn Martell, Kristina Duncombe Lowe, Daniel Le Grange, Jill Ehrenreich-May

**Affiliations:** 1 Center for the Treatment of Eating Disorders, Children’s Minnesota, Minneapolis, MN USA; 2 Department of Psychiatry, University of California, San Francisco, CA USA; 3 Department of Psychiatry and Behavioral Neuroscience, The University of Chicago, Chicago, IL USA; 4 Department of Psychology, University of Miami, Coral Gables, FL USA

**Keywords:** Avoidant/restrictive food intake disorder, Emotional disorders, Family-based treatment, Unified protocol, Transdiagnostic

## Abstract

**Background:**

This case report discusses the presentation and treatment of a nine-year-old female with a history of significant weight loss and food refusal using a combined approach of Family-Based Treatment (FBT) and the Unified Protocol for Transdiagnostic Treatment of Emotional Disorders in Children (UP-C).

**Case presentation:**

The patient was diagnosed with avoidant/restrictive food intake disorder (ARFID), separation anxiety disorder, and a specific phobia of choking, and subsequently treated with a modified version of FBT, in conjunction with the UP-C. At the end of treatment, improvements were seen in the patient’s weight and willingness to eat a full range of foods. Decreases in anxiety regarding eating/choking, fears of food being contaminated with gluten, and fears of eating while being away from parents were also observed.

**Conclusions:**

These findings highlight promising results from this combined treatment approach, referred to as FBT + UP for ARFID. Further research is needed to evaluate the use of this treatment in patients presenting with a variety of ARFID symptoms.

## Background

Avoidant/Restrictive Food Intake Disorder (ARFID), a complex and heterogeneous diagnosis, has been hypothesized along a dimensional model with presentations including sensory sensitivity, fear of aversive consequences, and lack of interest in eating [1, 2]. Significant literature exists on the treatment of pediatric feeding disorders supporting the use of behavioral feeding interventions among young children [3]. Recently, individual case reports/series have suggested other promising approaches for older children, adolescents, and adults with ARFID, using as a base either family-based treatment (FBT) [4–7];, cognitive behavioral therapy (CBT) [8–10];, or other novel approaches [11]. Despite these new approaches being studied, no published, randomized controlled trials have yet to evaluate their efficacy for the treatment of ARFID [2]. What appears to be lacking in the current treatment models is the ability to concurrently address the high rates of comorbid mood and anxiety disorders in patients with ARFID [12, 13], while also remaining focused on the medical complications associated with those patients who present underweight or exhibit significant nutritional deficiencies as part of this diagnosis. Consequently, this case presentation proposes a novel treatment approach that attempts to address both the psychological and emotional comorbidities associated in children and adolescents with ARFID, as well as the hallmark food avoidance features that appear across a heterogeneous array of presentations.

This case study describes the treatment of a patient with ARFID, using a combined approach of FBT [14] and the Unified Protocol for Transdiagnostic Treatment of Emotional Disorders in Children (UP-C) [15]. FBT + UP for ARFID was developed through a 3 year case consultation process with treatment developers of both FBT and the UP-C. Treatment focuses on a combination of techniques aimed at addressing both weight gain/normalization of eating and additional symptoms including fear, disgust, and worry or obsessive thoughts, as well as varying forms of functionally-related avoidance behavior and potential concomitant reinforcement of avoidance by parents/caregivers. A major advantage of this combined approach is that it allows the clinician to personalize treatment based on the patient’s specific presentation using a core set of evidence-based strategies and assessment tools (e.g., Top Problems [16];). The UP-C is transdiagnostic by definition, and contains evidence-based strategies that are flexible enough to address many of the maintaining symptoms that are unique to ARFID. There is also an adolescent version of the UP-C, which when combined with FBT makes this treatment model acceptable for a wide range of patients (named the Unified Protocol for Transdiagnostic Treatment of Emotional Disorders in Adolescents; UP-A). The UP for adults has previously been adapted for use with other eating disorder populations (anorexia nervosa, bulimia nervosa, and binge-eating disorder), with early results indicating improvments in anxiety sensitivity, experiential avoidance, and mindfulness [17].

While flexible, FBT + UP for ARFID always begins with sessions focused on FBT principles, including collaborative weighing, psychoeducation (specific to ARFID patients and their eating problems), family engagement, separating the eating problem from the child, charging parents with taking control of their child’s eating (including increasing volume and variety of foods), promoting weight gain as needed, and a family meal. The UP-C or UP-A is then added to build skills that empower the patient to cope with difficult emotions, address avoidance, and increase tolerance of emotions or disgust responses. The Unified Protocol for Transdiagnostic Treatment of Emotional Disorders (UP) [18] is an emotion-focused, evidence-based treatment that targets the core dysfunction of neuroticism in adults [19]. It has subsequently been adapted to address emotional disorders in youth with the development of the Unified Protocols for Transdiagnostic Treatment of Emotional Disorders in Children and Adolescents (UP-C and UP-A respectively [15];). These protocols bring together cognitive-behavioral techniques, such as cognitive reappraisal, problem-solving and opposite action strategies, including a variety of exposure paradigms and behavioral activation, as well as mindfulness techniques into a single treatment. The UP-C and UP-A present the same skills as the UP; however, the skills have been adapted to be developmentally sensitive in their presentation, as well as in their delivery. Furthermore, the UP-C and UP-A also target core emotional parenting behaviors that are common across emotional disorders in youth (i.e. high levels of criticism, over-control/over protection, inconsistency, and modeling of avoidance [15]). Research has provided support for the efficacy and feasibility of the UP, UP-A and UP-C for individuals with mood, anxiety, and other emotional disorders. The UP, in particular, has been shown to lead to significant improvements at post-treatment [20], as well as maintenance of gains at follow-up time points [21] . The UP-C was originally designed as a group version of the UP-A, with concurrent child and parent group content. However, the UP-C may be delivered in an individual therapy model and explicit directions for doing so are presented in the therapist guide. Preliminary evidence suggests the UP-C may be similarly effective to leading CBT approaches for childhood anxiety, with potential benefits for those youth with higher levels of parent-reported sadness, dysregulation or depressive symptoms [22, 23]. The UP-A has also been shown to improve symptoms of emotional disorders in adolescents. Results from multiple baseline, open-trial and initial wait-list controlled trial studies showed that adolescents evidenced significant improvement in their symptoms after receiving 16 sessions of treatment using the UP-A and gains were maintained at follow-up time points [24–26]. While results of initial patient outcomes for this combined FBT + UP for ARFID approach are not yet available (given this treatment is currently being studied as part of a larger, clinic-wide effectiveness study), feedback from individual patients and practitioners who have been trained in the model through a clinical teaching day at the Academy of Eating Disorders International Conference has been positive [27]. Consent to share the following case was provided by the family and patient. Changes in identifying information were made to protect patient privacy.

## Case presentation

“Laura” is a nine-year-old female, who presented with 38 lbs. of weight loss, poor oral intake, and medical instability in the context of fears about eating/choking secondary to a recent diagnosis of gluten intolerance. Ten months before she presented for treatment, Laura felt unwell after eating at a restaurant with her family. Following this experience, she became more anxious with eating, reporting frequent stomach aches and headaches. Laura’s family tried a variety of elimination diets, including stopping all dairy and gluten. Laura was seen multiple times by her pediatrician, who ultimately recommended allergy and celiac testing. Over the course of this time Laura lost 29% of her overall body weight. Laura’s symptoms continued to worsen, and she was eating little due to anxiety and a sensation of choking when eating. Parents noticed that her hair was falling out, her eyes appeared sunken, and she felt tired every day. She became increasingly afraid of separating from her parents, and her mother began getting calls from Laura’s school (3–4 times per day) due to frequent stomach aches or requests to see her mother.

Prior to presentation, Laura’s medical work-up showed focal chronic-type peptic duodenitis and reflux esophagitis. She was diagnosed with significant gluten sensitivity/intolerance, with a likely diagnosis of celiac disease. Laura had also been participating in weekly, individual play-based therapy for approximately 4 months to address her separation and other anxiety symptoms, without improvement. Her therapist did not have any expertise or experience in treating ARFID, therefore she was not focusing on weight regain or fears about eating. Laura was started on 20 mg of sertraline (liquid concentrate) 3 months prior to presentation at our service, though family had not seen any notable gains. Upon initial presentation to our team, Laura required hospitalization for 12 days for medical stabilization due to: symptomatic orthostasis, bradycardia, and severe malnutrition. During her hospital stay, Laura was diagnosed with ARFID, her sertraline was increased to 50 mg, and she was started on hydroxyzine, 5 mg TID to target pre-meal anxiety, nausea, and fullness. Following medical stabilization, Laura then began weekly outpatient treatment with her family to address the need for continued weight regain, anxiety/fears with eating, and separation anxiety. Given Laura had previously trended at or above the 85th percentile for BMI, the goal was to return her weight back to her personal healthy weight range.

The underlying assumption of FBT + UP for ARFID is that patients diagnosed with ARFID need a combination of treatment techniques that focus on both weight gain and/or normalizing eating while also addressing additional emotional disorder symptoms (i.e. anxiety, depression, obsessive-compulsive symptoms, emotional/situational avoidance). Patients and their parents begin with traditional FBT for several sessions (see Table 1 for content). Once progress with weight gain/regular eating are underway, the UP-C or UP-A modules are introduced. The UP-C has a flexible approach with core evidence-based principles and concurrent parenting content for emotional disorders that can be individualized for specific ARFID presentations [15]. Once the UP-C is added, the session breakdown continues as follows: 5 min weigh-in and update from patient on how eating is progressing, 30–40 min of individual therapy with the patient focused on the UP-C content, and 10–15 min with the patient and family to review session content, discuss how eating/weight gain are progressing, brainstorm challenges related to eating, and review homework/exposure practice. For younger patients, parents may be present for more/all of the session.

**Table 1:** FBT + UP-C for ARFID session content

Session	Content
*FBT Session 1*	Collaborative weighing, psychoeducation (specific to ARFID patients), separating the eating problem from the child, charging parents with taking control of their child’s eating, and beginning the discussion of utilizing rewards.
*FBT Session 2*	Engage family in family meal to further assess patient’s eating, address any mealtime behaviors that are getting in the way of success, and work to empower parents to begin helping their child make changes to their eating.
*FBT Sessions 3+*	For very underweight patients, additional FBT sessions focus on building the parental alliance and discussing ways to improve the parent’s ability to work together on the task of weight gain and related symptoms (food avoidance, anxieties around eating, etc). For patients who are not underweight or are gaining weight appropriately, the UP session content may begin to be added.
*FBT + UP-C Module 1: Introduction to the Unified Protocol for the Treatment of Emotional Disorders in Children*	Introduces child/parents to the treatment model/structure and describes the CLUES skills (Consider how I feel, Look at my thoughts, Use detective thinking and problem solving, Experience my feelings, Stay healthy and happy), discusses the purpose of emotions and begins to build emotional awareness, and identifies top problems and treatment goals. Top problems may focus on ARFID related goals or be more wide-range to address other emotional avoidance or related diagnoses.
*FBT + UP-C Module 2: Getting to Know Your Emotions*	Learn to identify and rate intensity of different emotions, normalizes emotional experiences, discusses the three parts of the emotional experience and the cycle of avoidance, explains true/false alarms, and identifies rewards for new behaviors.
*FBT + UP-C Module 3: Using Science Experiments to Change our Emotions and Behavior*	Learn about the concept of “acting opposite” and using science experiments to help with acting opposite/emotional behaviors, explains the connection between activity and emotion and assigns emotion and activity tracking as an experiment.
*FBT + UP-C Module 4: Our Body Clues*	Describe the concept of body clues and their relation to strong emotions, learn to identify body clues for different emotions, teach the skill of body scanning to develop awareness of body clues, help child practice experiencing body clues without using avoidance/distraction through interoceptive exposures.
*FBT + UP-C Module 5: Look at my Thoughts*	Introduce the concept of flexible thinking and teach children to recognize common “thinking traps.”
*FBT + UP-C Module 6: Use Detective Thinking*	Introduce and apply detective thinking.
*FBT + UP-C Module 7: Problem Solving and Conflict Management*	Introduce and apply problem solving, discuss use of problem solving for interpersonal conflicts or challenges related to eating.
*FBT + UP-C Module 8: Awareness of Emotional Experiences*	Teach children about present moment awareness, introduce non-judgmental awareness- especially with relation to strong disgust responses.
*FBT + UP-C Module 9: Introduction to Emotion Exposure*	Review skills learned to date in the UP-C, review the concepts of emotional behaviors and “acting opposite” in preparation for a new type of science experiment called “exposure,” complete a demonstration of an exposure using a toy or other object, work together with child and parents to begin developing plans for upcoming exposures.
*FBT + UP-C Module 10: Facing Our Feelings – Part 1*	Review the concept of using science experiments to face strong emotions, introduce the idea of safety behaviors and subtle avoidance behaviors (e.g., distraction), practice a science experiment to face strong emotions (sample situational emotion exposure), make plans for future science experiments for facing strong emotions (individualized situational emotion exposures).
*FBT + UP-C Module 11: Facing Our Feelings – Part 2*	Plan and execute initial situational emotion exposure in session, plan and execute additional situational emotion exposure activities in future sessions and at home.
*FBT + UP-C Module 12: Wrap Up and Relapse Prevention*	Review Emotion Detective skills learned in the UP-C program, plan for facing strong emotions in the future, celebrate progress made in treatment program, create a plan for sustaining and furthering progress after treatment, distinguish lapses from relapses and help family recognize warning signs of relapse.

As illustrated in Table 2, over the course of treatment Laura’s weight increased from 36.7 kg to 44.7 kg (percent goal weight from 81.4 to 91.4%), with family noting significant improvements in energy level and ability to participate in school and other physical activities. During initial FBT sessions, the focus was on weight gain using foods that Laura felt were safe and could allow her to regain weight efficiently. In session two, a family meal was completed, where the therapist worked to separate the illness from Laura and decrease blame (see FBT manual [14]), as well as discuss rewards that could be utilized to encourage Laura to challenge herself with eating. After two sessions of FBT (and with Laura’s weight increasing), the UP-C was added to sessions, though the focus of each subsequent session also remained on weight regain and parental support/empowerment. Of note, Laura’s family took to the principles of FBT quickly, but continued to benefit from each session’s focus on graphing the patient’s weight, problem solving any challenges during weeks where weight was stable or down, and empowering parents to work closely together on how to best refeed their daughter.

**Table 2:** Top problems and weight

	Baseline	End of treatment	5 months post treatment
Top Problems (Parent)			
Fear of choking/eating fear foods	8	3	2
Being away from mother/eating away from mother	8	2	2
Sleeping alone	7	2	0–1
Top Problems (Child)			
Fear of choking/eating fear foods	8	3	1
Being away from mother/eating away from mother	8	2	2–3
Sleeping alone	8	0	0
Weight Presentation			
Kilograms	36.7	44.7	50.4
BMI %ile	41.3	70.7	81.2
% Goal Weight	81.4	91.4	97.1

Top Problems were rated 0–8, with 0 being not a problem and 8 being very much a problem. BMI %ile = Body Mass Index Percentile. % Goal Weight = Percentage of treatment goal weight utilizing the 85th percentile for Body Mass Index

The patient and family identified three Top Problems (an ideographic assessment tool by Weisz et al. [16] modified for use in the UP-C and UP-A by Ehrenreich-May et al. [15]) they wanted to address in treatment including: 1) decrease fears of choking/eating feared foods, 2) be away from/eat away from mother, and 3) patient sleeping in her own bed again. Additionally, the therapist reinforced an overarching goal of Laura returning to a healthy weight range as crucial for her recovery. All subsequent treatment sessions involved reviewing Laura’s weight/eating, teaching content from the UP-C modules, and discussing home learning assignments.

As treatment progressed and the patient learned skills to better manage her emotions, she became more willing to try foods that she was avoiding. With the help of the treating clinician, Laura created an exposure hierarchy with numerous feared foods and situations (e.g. meats, pasta, nuts, eating with adults other than her mother, eating at restaurants, being away from her mother, and sleeping in her own bed). Because Laura’s fears of eating most foods were greatly impacting her overall functioning, the therapist chose to move up exposure work after introducing the three parts of the emotional experience, discussing the cycle of avoidance, and describing true/false alarms. During the exposure work, Laura created a ladder to break down the steps of each exposure, beginning with simply describing the food in a non-judgmental way and later touching, licking, taking a tiny bite, and eventually taking larger bites of these foods. Each of these skills were taught to Laura using specific content from the UP-C. Laura and her parents were encouraged by her success and began implementing exposures outside of sessions.

Laura continued to add more new foods at home and was able to attempt other types of foods in session. Once in-session exposures became easier for Laura, the therapist had her add interoceptive exposures (e.g. running in place), while also eating feared foods to attempt to evoke increased feelings of anxiety and simulate a more naturalistic experience of distress. As therapy progressed, Laura began eating at restaurants again, as well as in more situations away from her mother (e.g., church, school cafeteria). She was able to stop the use of hydroxyzine, but continued on her sertraline. Treatment ended when Laura returned to eating nearly all foods, in numerous settings (school lunchroom, other’s homes) away from her mother, and family felt able to manage remaining avoidance (e.g. working on eating at a greater variety of restaurants while away from their hometown). Laura had also regained weight to the 71st percentile for BMI (91.4% of her previously healthy weight range), and her parents felt fully equipped in their ability to continue helping her restore weight. Laura completed 29 sessions over the course of 10 months of weekly or biweekly therapy.

## Discussion and conclusions

This case study illustrates that the FBT + UP for ARFID therapy model, which combines and modifies previously developed evidence-based treatments, was feasible and helpful in allowing this patient to gain weight, return to eating a diverse range of foods in a variety of settings, and decrease anxiety about eating/being away from her mother. Notably, when this family returned for a follow-up 5 months after completing treatment, the patient’s weight had continued to increase (50.4 kg/81st percentile for BMI/97.1% of goal weight), she had started menstruating, and she was able to separate and eat apart from her mother without significant difficulty. The patient and parents also rated her fears of choking and eating previously feared foods as 1 and 2’s on an 8-point likert scale (see Table 2).

This patient was a good treatment candidate for FBT + UP for ARFID given she endorsed significant anxiety prior to treatment and also met criteria for several concurrent anxiety disorder diagnoses. Another major benefit of the treatment is the ability to flexibly offer the various modules that may benefit each patient based on their specific needs and ARFID presentations. For example, this patient benefited from exposure work, learning non-judgmental awareness, and improving awareness of physical sensations, while other patients may need more focus on cognitive reappraisal, problem-solving, and other types of opposite action [15]. Additionally, given Laura had lost a significant amount of weight she required a treatment that also focused on weight restoration as one of its core principles. A major advantage of this combined treatment approach is the ability for clinicians to tailor each session to the specific needs of their individual patient, including returning to solely FBT sessions if weight gain or nutritional dificiencies are not progressing appropriately.

While several novel approaches for the treatment of ARFID have been suggested [7, 10, 11], randomized control trials have yet to be presented regarding their efficacy. Even with some intervention research aiming to address the heterogeneous symptoms of ARFID, no treatment to date has proposed a model that addresses both the varied presentations of ARFID, as well as its full range of common comorbid disorders, in one cohesive approach that is flexible and adaptable to the individual. While the development of symptom specific treatment approaches to ARFID is logical, it does not address the heterogeneous nature of this disorder and can impede dissemination [28]. With so many different presentations of ARFID and high rates of comorbid disorders, one clear treatment that can be used flexibly to adapt to the range of ARFID presentations and co-occurring disorders would provide an efficient and cohesive approach to treating youth with ARFID. Further examination of FBT + UP for a wide-range of ARFID presentations among youth continues. A study to establish an ideal combination of FBT and UP strategies for youth with ARFID between the ages of 6–18 years, and the preliminary efficacy of this approach, is a next logical step in this research.

Finally, some limitations with this case study should be noted. First, it was not possible to ascertain whether FBT in isolation would have worked as effectively for this patient as this combined FBT + UP-C approach. While anxiety reduction has been shown in nutritional-based therapies, such as FBT, it is unclear if patients with profound phobic and other concurrent anxiety would benefit as greatly without specific skills and exposure work inherent in the UP-C. Additional limitations of this case study include the absence of objective assessment of psychological outcomes. That said, this young person made significant improvements in terms of weight, both at completion of treatment and at follow-up. Moreover, *Top Problems* rating by both the patient and parents also appear to indicate meaningful improvements in a variety of behavioral domains. However, without objective measures it is difficult to ascertain whether anxiety reduction allowed for behavioral change, or whether behavioral change caused anxiety reduction over the course of the UP-C. Future studies should attempt to parce out when and for whom this combined treatment approach is most effective.

## Data Availability

All authors had access to the relevant material in the generation and review of this manuscript. Due to ethical concerns, supporting data cannot be made openly available.

## Abbreviations

ARFID: Avoidant/Restrictive Food Intake Disorder
FBT: Family Based Treatment
FBT+UP: Family Based Treatment with the Unified Protocol
Kg: Kilograms
TID: Three times a day
UP: Unified Protocol for Transdiagnostic Treatment of Emotional Disorders
UP-A: Unified Protocol for Transdiagnostic Treatment of Emotional Disorders in Adolescents
UP-C: Unified Protocol for Transdiagnostic Treatment of Emotional Disorders in Children
